# Local treatment with ascorbic acid accelerates recovery of post-sutured Achilles tendon in male Wistar rats

**DOI:** 10.1590/1414-431X20198290

**Published:** 2019-09-02

**Authors:** M. Souza, S.A.S. Moraes, D.R. de Paula, A.A. Maciel, E.J.O. Batista, D.G.F. Silva, C.P. Bahia, K.R.H.M. Oliveira, A.M. Herculano

**Affiliations:** 1 Laboratório de Neurofarmacologia Experimental, Instituto de Ciências Biológicas, Universidade Federal do Pará, Belém, PA, Brasil; 2 Instituto de Ciências da Saúde, Universidade Federal do Pará, Belém, Pará, Brasil; 3 Núcleo de Medicina Tropical, Universidade Federal do Pará, Belém, Pará, Brasil

**Keywords:** Tendon recovery, Tenotomy, Ascorbic acid, Tendon suture

## Abstract

Tendon rupture is a very frequent accident involving average people and high-performance athletes. Clinical studies describe tendon recovery as a painful and slow process involving different biochemical and histological events. Ascorbic acid (AA) is a potent antioxidant as well as an important cofactor for collagen synthesis. In the current study, we evaluated if local treatment with AA is able to promote tendon repair in tenotomized rats. Animals were submitted to Achilles tendon rupture followed by surgical suture. Control and AA groups received *in loco* injection of saline solution (0.9% NaCl) and 30 mM AA, respectively. Histological and functional recovery of Achilles tendon tissue was evaluated at 7, 14, and 21 days post-surgery. Hematoxylin/eosin staining and collagen fluorescence analysis showed intense disarrangement of tendon tissue in the saline group. Tenotomized animals also showed hypercellularity in tendon tissue compared with non-tenotomized animals. The Achilles functional index (AFI) showed a significant decrease of tendon functionality in tenotomized animals at 7, 14, and 21 days post-surgery. AA accelerated tissue organization and the recovery of function of the Achilles tendons. The beneficial effect of AA treatment was also observed in the organization of the collagen network. Data presented in the current work showed that *in loco* treatment with AA accelerated the recovery of injured Achilles tendon post-surgery.

## Introduction

Tendon ruptures represent more than fifty percent of tendon injuries recorded in average people. Surgical procedure involving tendon suture is the first clinical approach in tenotomized patients. Tissue recovery is characterized by chronic pain and progressive loss of muscle function. Clinical studies also describe that patellar tendon, wrist extensors tendon, and Achilles tendon are the main target of tendinopathies and ruptures (1–3).

The tendon is a connective tissue with fibrous-elastic texture constituted by a dense collagen network. Tenocytes are a kind of fibroblast present in tendon tissue that are responsible for the synthesis of collagens and other components of tendons such as proteoglycans, glycoproteins, and elastin (4,5). Activation of tenocytes after tendon rupture are essential for tissue repair. In fact, tendon recovery starts with the inflammatory period, which is characterized by leucocyte migration to the injured tissue and intense collagen degradation. This phase is followed by tenocyte proliferation and an increase in collagen synthesis (6). The last phase of tissue repair is characterized by metabolism of extracellular matrix as well as by intense remodeling of collagen fibers into the injured tissue (7). All these histological phases are evolutionarily conserved in mammalians and thus animal models have emerged as a powerful tool to evaluate tendon recovery (8).

Overproduction of reactive oxygen species (ROS) is a biochemical event observed in the different phases of tissue recovery (9). ROS interactions with collagen proteins decelerate the formation of the collagen network and consequently retard tissue remodeling (10). In other words, intense ROS production in injured tendons is a harmful event for tendon recovery.

Ascorbic acid (AA) is a well-known ROS scavenger in biological systems. In addition, it is widely described that AA acts as a cofactor for synthesis of collagen proteins (11 [Bibr B12]). Systemic treatment utilizing different concentrations of AA has been previously tested in an animal model of tenotomy. However, the effectiveness of these systemic treatments is variable and contradictory in the literature (11–13). Our hypothesis was that low vascularization of tendon tissue hinders the pharmacological action of AA during systemic treatment. Based on this hypothesis, the current work evaluated if local treatment with AA is able to accelerate tissue recovery of Achilles tendon of tenotomized rats.

## Material and Methods

### Animals and surgical protocol

Male Wistar rats (250–300g) were provided by animal facilities of the Federal University of Pará (UFPa, Brazil). Animals were kept in polypropylene cages at 25°C in a controlled dark/light cycle (12:12) with food and water *ad libitum*. All experimental procedures were previously approved by the ethical committee for care and use of laboratory animals from UFPa (Protocol number 161-13). Animals were anesthetized by intraperitoneal injection of ketamine (80 mg/kg)/xylazine (12 mg/kg) solution. The tibia region of the right paw was manually trichotomized and a longitudinal skin incision (about 0.5 cm) was made to access the Achilles tendon. Briefly, the Achilles tendon was transected in an axial fashion at 0.5 cm from its calcaneal insertion followed by Kessler suture as previously described by Murrell et al. (14). All these steps were followed by local asepsis and skin suture using 4.0 nylon monofilament.

After surgical treatment, the animals were kept in cages without movement restriction or immobilization of legs. The post-anesthetic recovery of the animals was monitored for 2 h, with no intercurrence. For this, the maintenance of the vital signs and the return of the mobility of the animals were observed.

Animals were subdivided into control group (CTRL, n=6); vehicle group (VEH, n=6), which was submitted to tendon rupture followed by surgical suture, and post-treated *in loco* with saline solution (0.9%); ascorbic acid group (AA, n=6, which was submitted to tendon rupture, surgical suture, and posteriorly treated *in loco* with ascorbic acid (30 mM). All treatments were administrated into the paratendinous region with a 26-gauge needle once every two days after injury until day 12 or 20 of saline solution or ascorbic acid injection in a volume of 40 µL (15).

### Histological analysis

Achilles tendons of control and experimental groups were evaluated at 14 and 21 days post-tendon rupture. Tendon tissues were quickly dissected and fixed in 4% paraformaldehyde during 12 h as described by Moraes et al. (15). Briefly, tendons were washed three times with 0.1 M phosphate buffer, pH 7.4, and cryoprotected by sequential immersion in a gradient of sucrose solution (10, 20, and 30%). Longitudinal sections (20 μm) of tendon tissues were stained with hematoxylin-eosin (HE) and cover-slipped using Permount^®^ (Fisher Scientific, USA). The images were visualized and recorded utilizing a light microscope (Nikon, Eclipse E800, Japan) and analyzed by ImageJ^®^ software (NIH, USA).

### Determination of cell number

The total number of cells in tendons was measured by a direct count of DAPI-stained nuclei. Longitudinal sections of the tissue were washed one time with distilled water for 5 min and permeabilized using 0.1% Triton X-100 for 10 min. Tissue sections were incubated with DAPI solution (1:10,000) for 1 min and mounted on glass slides with N-propyl-gallate. Cell nuclei were analyzed by fluorescent microscopy at 420 nm. The number of cells was determined by the double-blind count in 3 random areas (0.830 mm^2^) of tendon sections.

### Collagen fluorescence

Collagen organization in tendons was evaluated by use of collagen auto-fluorescence as previously described by Hoell et al. (16). Longitudinal sections (20 µm) were incubated for 30 s with eosin solution. Proximal, medial, and distal regions of tendon tissue were evaluated by fluorescence microscopy with a standard filter for fluorescein isothiocyanate (FITC). Images were recorded and analyzed using fluorescence microscopy and ImageJ^®^ software.

### Achilles functional index

Tendon functionality was measured utilizing Achilles functional index (AFI) as proposed by Murrell et al. (20). Control and experimental animals had their hindpaws painted with nontoxic blue ink and then the animals were placed in the walkway apparatus (10×60 cm) leaving their footprints on a white paper. The animal footprints were recorded, digitalized, and evaluated using the ImageJ software. The values of footprint length (FL), foot spreading (FS), and the intermediary test factor (IFT) were applied in the followed equation of Achilles functional index: AFI = 74 (FL) + 161 (FS) + 48 (ITF) – 5.

### Statistical analysis

All data are reported as means±SD. Comparison among groups was evaluated using analysis of variance (ANOVA) followed by Tukey’s post-test. P<0.05 was considered significant and all statistical tests were performed using BioEstat 5.2 Software (Mamirauá Institute, Brazil).

## Results

### AA treatment induced histological improvement in injured tendon tissue

**Figure 1:**
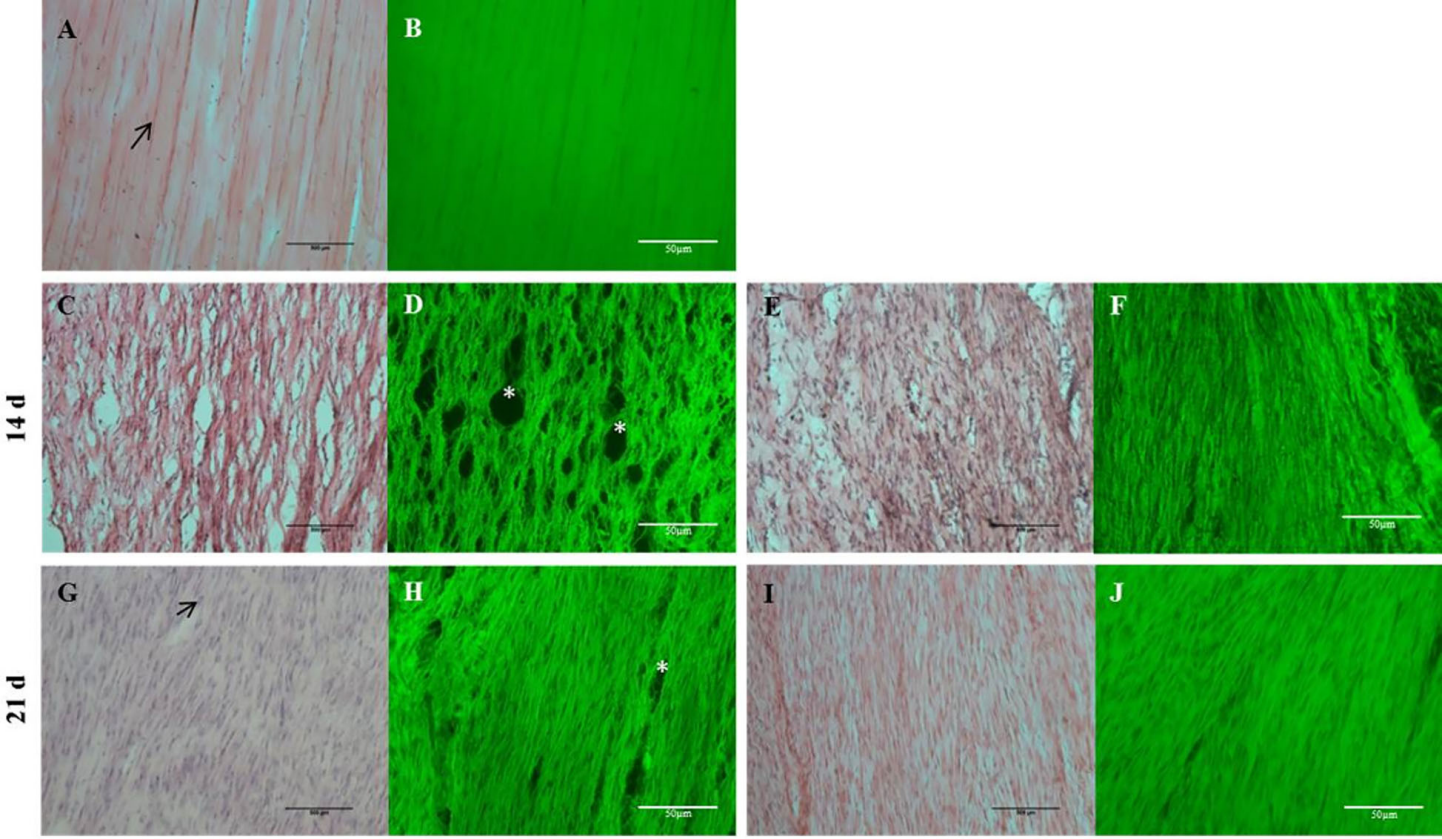
Hematoxylin-eosin staining and fluorescence microscopy (green) of longitudinal sections of rat Achilles tendon at 14 and 21 days post-surgery. Control group (**A** and **B**), vehicle group analyzed at 14 (**C** and **D**) and 21 days post-surgery (**G** and **H**), and ascorbic acid group analyzed at 14 (**E** and **F**) and 21 days post-surgery (**I** and **J**) are shown. *Tissue holes. Scale bar, 50 μm.

Histological analyses of Achilles tendon showed intense tissue disarrangement in tenotomized animals compared with control (Figure 1). Tendons of the vehicle group presented numerous tissue holes at 14 and 21 days post-surgery (Figure 1C and G). Tenotomized animals that received local injection of AA showed better tissue organization as well as a decreased number of tissue holes compared with vehicle group (Figure 1C and E).

**Figure 2:**
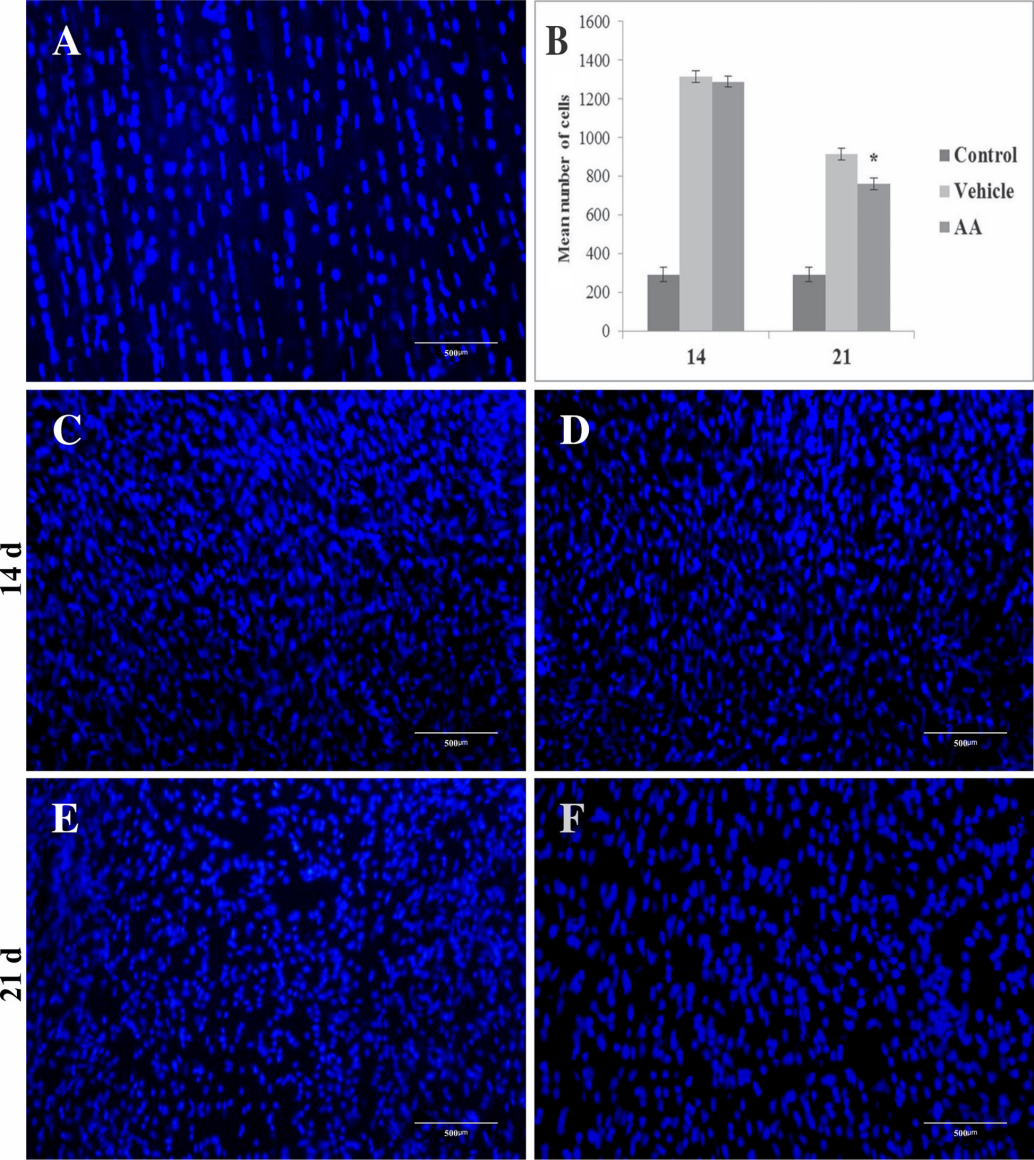
Photomicrographs of DAPI staining in tendon tissue of control group (**A**), vehicle group at 14 (**C**) and 21 days post-surgery (**E**), and ascorbic acid (AA) group at 14 (**D**) and 21 days post-surgery (**F**). Cell number data are reported as means±SD for n=3 (**B**). *P<0.01 *vs* vehicle (ANOVA and Tukey’s post-test). Scale bar: 500 μm.

Autofluorescence of collagen proteins demonstrated intense disarrangement of the collagen network in the injured tendon at 14 and 21 days post-surgery. Our data also showed that ruptured tendons treated with AA presented better organization of the collagen network (Figure 1B, D, F, H, and J). The measurement of cell number in tenotomized tissue demonstrated hypercellularity at 14 and 21 days post-surgery (Figure 2C and E). Treatment with AA did not prevent this increase in cell number induced by tissue rupture at 14 days post-surgery. However, tendon tissue treated with AA presented a decrease in the number of cells at 21 days post-surgery compared with the vehicle group (Figure 2B, D, and F).

### AA treatment induced functional improvement in tenotomized rats

**Figure 3:**
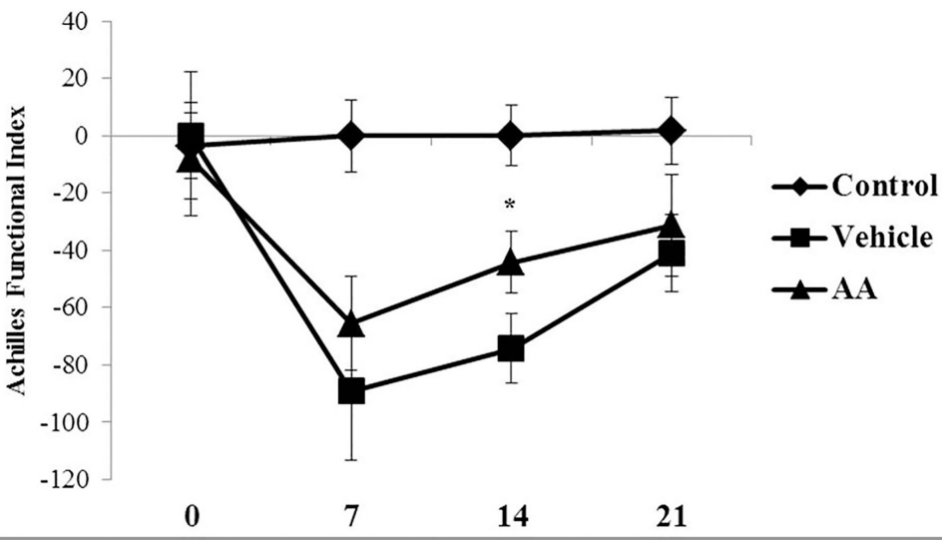
Achilles functional index was measured at 0, 7, 14, and 21 days post-surgery in control, vehicle, and ascorbic acid (AA) groups. Data are reported as means±SD for n=6. *P<0.05 *vs* vehicle (ANOVA and Tukey’s post-test).

Tenotomized animals presented a significant decrease in AFI values compared with non-tenotomized animals at 7 days post-surgery (vehicle: −89.2±23 *vs* Control: −2.7±12) (Figure 3). AFI values of tenotomized animals increased progressively at 14 (vehicle: −74.2±11) and 21 (vehicle=−41±13) days post-surgery. Local treatment with AA attenuated the intense functional impairment induced by tendon injury at 7 (vehicle: −89.2±23 *vs* AA: −65±16) and 14 days post-surgery (vehicle: −74.2±11 *vs* AA −44±10). No statistical difference was observed between AFI values of the AA group and the vehicle group at 21 days post-surgery (Figure 3).

## Discussion

Surgical suture of Achilles tendon is the first clinical procedure performed in patients after total tendon rupture. Clinical observations describe that histological and functional recovery of tendons are variable and very painful (13,17). Based on this report, the development of new strategies for the treatment of injured tendons is very important to minimize the suffering of tenotomized subjects. In the current study, we demonstrated for the first time that *in loco* treatment with AA accelerated histological and functional recovery of ruptured Achilles tendons.

Histological events related to tendon repair start with the intense inflammatory period, which is followed by proliferative and remodeling phases. These histological events are evolutionarily conserved in mammals and, based on this, murine models have emerged as a useful tool for the development of new treatments for tendon recovery (6,8).

As previously described, inflammatory activation on tendon tissue is mediated by cytokines release, cell phagocytosis, and overproduction of ROS (9,18). Data from the literature also describe that ROS production inhibits synthesis and polymerization of collagen in injured tendons (10,19,20). In agreement to this, our group has previously described that local inhibition of nitric oxide synthase (NOS) accelerates tendon recovery in tenotomized rats (15). The positive effect of the NOS inhibitor on tendon repair demonstrates that nitric oxide (NO) negatively regulates tissue recovery. Our findings are in agreement with all these results, which suggest that *in loco* oxidative stress is harmful to the process of tendon recovery (21,22).

It is well-known that complete disarrangement and intense cell proliferation occurs in injured tendons in the first week post-lesion (23,24). These alterations are progressively decreased from the proliferative to the remodeling period of tendon recovery (25). Data presented in the current study are in agreement with these findings and support the use of animal models for studies aimed to describe histological events related with tendon recovery. It was also observed that AA was not able to prevent tenotomy-induced hypercellularity in the first period of tendon recovery. However, animals treated with AA had a decreased number of cells in injured tendons compared with non-treated animals at 21 days post-surgery (Figure 2F).

The current study showed for the first time that local treatment with AA exerted beneficial effects in injured tendons of rats. Our data were supported by cellular, histological, and functional improvements observed in treated animals. Although posterior studies need to be performed to clarify the molecular mechanisms elicited by AA on injured tendons, its action as a co-factor for collagen synthesis and potent antioxidant seems to be important. Taken together, our findings support that local treatment with AA could represent a future adjuvant to accelerate the recovery of injured tendons.

## References

[1] Lipman K, Wang C, Ting K, Soo C, Zheng Z (2018). Tendinopathy: injury, repair, and current exploration. Drug Des Devel Ther.

[B02] Longo UG, Ronga M, Maffulli N (2009). Achilles tendinopathy. Sport Med Arthrosc Rev.

[3] Rees JD, Maffulli N, Cook J (2009). Management of tendinopathy. Am J Sports Med.

[4] Tresoldi I, Oliva F, Benvenuto M, Fantini M, Masuelli L, Bei R (2013). Tendon's ultrastructure. Muscles Ligaments Tendons J.

[5] Kannus P (2000). Structure of the tendon connective tissue. Scand J Med Sci Sport.

[6] Sharma P, Maffulli N (2006). Biology of tendon injury: healing, modeling and remodeling. J Musculoskelet Neuronal Interact.

[7] Muller SA, Todorov A, Heisterbach PE, Martin I, Majewski M (2015). Tendon healing: an overview of physiology, biology, and pathology of tendon healing and systematic review of state of the art in tendon bioengineering. Knee Surg Sport Traumatol Arthrosc.

[8] Thomopoulos S, Parks WC, Rifkin DB, Derwin KA (2015). Mechanisms of tendon injury and repair. J Orthop Res.

[9] Bestwick CS, Maffulli N (2004). Reactive oxygen species and tendinopathy: do they matter?. Br J Sports Med.

[10] Murrell GAC (2007). Oxygen free radicals and tendon healing. J Shoulder Elb Surg.

[11] Du J, Cullen JJ, Buettner GR (2012). Ascorbic acid: chemistry, biology and the treatment of cancer. Biochim Biophys Acta.

[B12] Lima C, Pereira A, Silva J, Oliveira L, Resck M, Grechi C (2009). Ascorbic acid for the healing of skin wounds in rats. Braz J Biol.

[13] Chen Q, Espey MG, Sun AY, Lee J, Krishna MC, Shacter E (2007). Ascorbate in pharmacologic concentrations selectively generates ascorbate radical and hydrogen peroxide in extracellular fluid in vivo. Proc Natl Acad Sci USA.

[14] Murrell GA, Lilly EG, Davies H, Best TM, Goldner RD, Seaber AV (1992). The achilles functional index. J Orthop Res.

[15] Moraes SA, Oliveira KR, Crespo-López ME, Picanço-Diniz DL, Herculano AM (2013). Local NO synthase inhibition produces histological and functional recovery in Achilles tendon of rats after tenotomy: Tendon repair and local NOS inhibition. Cell Tissue Res.

[16] Hoell T, Huschak G, Beier A, Huttmann G, Minkus Y, Holzhausen HJ (2006). Auto fluorescence of intervertebral disc tissue: a new diagnostic tool. Eur Spine J.

[17] Docheva D, Müller SA, Majewski M, Evans CH (2015). Biologics for tendon repair. Adv Drug Deliv Rev.

[18] Meier B, Radeke HH, Selle S, Younes M, Sies H, Resch K (1989). Human fibroblasts release reactive oxygen species in interleukin-1 or tumour necrosis factor-alpha. Biochem J.

[19] Lin J, Wang MX, Wei A, Zhu W, Murrell GA (2001). The cell specific temporal expression of nitric oxide synthase isoforms during Achilles tendon healing. Inflamm Res.

[20] Murrell GAC, Szabo C, Hannafin JA, Jang D, Dolan MM, Deng X (1997). Modulation of tendon healing by nitric oxide. Inflamm res.

[21] Radák Z, Naito H, Kaneko T, Tahara S, Nakamoto H, Takahashi R (2002). Exercise training decreases DNA damage and increases DNA repair and resistance against oxidative stress of proteins in aged rat skeletal muscle. Pflugers Arch.

[22] Morikawa D, Itoigawa Y, Nojiri H, Sano H, Itoi E, Saijo Y (2014). Contribution of oxidative stress to the degeneration of rotator cuff entheses. J Shoulder Elbow Surg.

[23] Bring DK, Kreicbergs A, Renstrom PA, Ackermann PW (2007). Physical activity modulates nerve plasticity and stimulates repair after achilles tendon rupture. J Orthop Res.

[24] Riley G (2008). Tendinopathy--from basic science to treatment. Nat Clin Pract Rheumatol.

[25] Korntner S, Kunkel N, Lehner C, Gehwolf R, Wagner A, Augat P (2017). A high-glucose diet affects Achilles tendon healing in rats. Sci Rep.

